# Function of Mitogen-Activated Protein Kinases in Hepatic Inflammation

**Published:** 2021

**Authors:** Gabrielle Westenberger, Jacob Sellers, Savanie Fernando, Sadie Junkins, Sung Min Han, Kisuk Min, Ahmed Lawan

**Affiliations:** 1 Department of Biological Sciences, University of Alabama in Huntsville, Huntsville, Alabama 35899, USA; 2 Division of Kinesiology, University of Texas at El Paso, El Paso, Texas 79968, USA; 3 Department of Aging and Geriatric Research, Institute of Aging, College of Medicine, University of Florida, Gainesville, Florida 32610, USA

**Keywords:** MAP kinase, Hepatic inflammation, Obesity, Liver injury

## Abstract

The western diet and overuse of anti-inflammatory medication have caused a great deal of stress on the liver. Obesity and the associated inflammatory state in insulin-responsive tissues result in the release of pro-inflammatory cytokine that activates the stress-responsive MAPKs, p38 MAPK, and JNK. These MAPKs have figured prominently as critical effectors in physiological and pathophysiological hepatic inflammation. In contrast, evidence for a role for ERK1/2 in hepatic inflammation has been less well developed. In this review article, we describe recent insights into the physiology and pathophysiology of the role of stress-responsive MAPKs in hepatic inflammation during obesity and liver injury with a focus on macrophages, hepatocytes and hepatic stellate cells. In response to metabolic stress and liver injury, JNK activation in macrophages and hepatocytes promotes the secretion of inflammatory cytokines and macrophage and neutrophil infiltration. p38 MAPK plays an important role in contributing to the progression of hepatic inflammation in response to various hepatic cellular stresses, although the precise substrates mediating these effects in hepatocytes and hepatic stellate cells remain to be identified. Both JNK and p38 MAPK promotes profibrotic behavior in hepatic stellate cells.

## Introduction

The liver is commonly regarded as the gatekeeper of good health. The liver is composed of many different cells including hepatocytes, cholangiocytes, hepatic stellate cells and Kupffer cells, among others [1]. Hepatocytes make up the majority of the liver’s cell population and thus are responsible for performing many of the functions attributed to the liver. These functions include the breakdown of foreign compounds like alcohol, blood volume regulation, lipid and cholesterol homeostasis, immune and endocrine system support and drugs metabolism [1].

The western diet and overuse of anti-inflammatory medication have caused a great deal of stress on this vital organ. In 2019, approximately two million deaths worldwide were due to liver disease [2]. The liver is known to be very resilient, as it is constantly exposed to antigens and exotoxins presented from the food in the intestine. With this being said, when the liver undergoes damage either physically or chemically, the local immune system composed of natural killer cells and Kupffer cells release cytokines that play a role in the adaptive response and promote the eradication of harmful pathogens [3]. However, lipid accumulation can cause unwarranted damage to the delicate system and induce liver inflammation [4]. Acetaminophen (APAP), a widely used analgesic, overdose is a leading cause of drug-induced hepatoxicity. APAP causes acute liver failure through oxidative stress, hepatocyte apoptosis and necrosis [5].

## Inflammation and Hepatic Dysfunction

Inflammation can be defined as restoration of functionality in response to infection, tissue injury and stress [13]. Inflammation is no longer limited to autoimmune infections, it is now regarded as a symptom of chronic diseases [6]. Hepatic inflammation is linked with common acute and chronic liver diseases. It is known that high levels of fatty acids (FAs), particularly saturated FAs like palmitate, stimulate hepatoxicity and metabolic dysfunction are key contributors to hepatic inflammation [5]. The enhanced inflammatory signaling is a major driver of more advanced diseases of the liver, including nonalcoholic steatohepatitis (NASH). The innate immune system is the first line of defense against foreign pathogens. Toll like receptors (TLRs) are a family of nonclonal, germline- encoded pattern recognition receptors [7]. In humans, there are 10 different functional TLRs that recognize pathogen associated molecular patterns (PAMPs) and damage associated molecular patterns (DAMPs). PAMPs are highly conserved molecules expressed by invading pathogens, whereas DAMPS are endogenous components of dying or damaged cells [7]. The liver is under constant exposure to gut derived microbiota; therefore, it is important that TLR signaling pathways are at lower levels compared to other organs. The stimulation of TLRs with a ligand activates downstream adaptor molecules like Interleukin-1 receptor. This triggers a signaling cascade that converges on MAPKs, nuclear factor-κB (NF-κB), and interferon response (IFN) factors resulting in the transcription of proinflammatory cytokines like IL-6 and tumor necrosis factor alpha (TNF-α) [8]. Recently, it has been suggested that TLRs play a significant role in the pathogenesis of many liver diseases. For example, studies have indicated that there is a significant contribution of TLR4 in the damaging effects of alcoholic liver disease (ALD). Liver inflammation and induced liver fibrosis is stimulated by Kupffer cells when bound to TLR4 allowing for the production of pro-inflammatory cytokines and profibrogenic factors [8].

Monocyte Chemoattractant Protein- 1 (MCP-1) is an important chemokine that activates macrophages and pro-inflammatory cytokines. Macrophages are divided into two phenotypes: M1 and M2. The M1 phenotype works to promote pro-inflammatory cytokines like TNF-α and IL-1β which contribute to the pathogenesis of hepatic steatosis. The M2 phenotype contributes to insulin sensitivity and inhibits inflammation [9]. TNF-α induces apoptosis of hepatocytes, whereas IL-6 can be protective or damaging based on the target cell.

## Mitogen-Activated Protein Kinases (MAPKs)

MAPKs are a family of serine/threonine kinases and have the ability to regulate cellular processes by transmitting extracellular stimuli to intracellular responses [10–12]. In the liver, MAPKs play an important role in regulating processes that control inflammation. MAPKs are activated through a three tiered “core signaling module” [11] MAPK activation can start with a variety of different external stimuli including the binding of a ligand to a receptor tyrosine kinase, cytokine receptor, or G-protein coupled receptor (GPCR) among others [11]. This event catalyzes the activation of a family of protein kinases known as MAPK - kinase- kinase (MAP3Ks). MAP3Ks are responsible for phosphorylating a family of dual specificity kinases called MAPK/extracellular signal regulated kinases (MEKs or MKKs). This phosphorylation occurs at a conserved Ser/Thr site [11,13]. Finally, the activation of MAPKs is catalyzed by MKKs at a conserved Thr- X- Tyr motif which allows for MAPK to induce the appropriate response [13]. This mechanism allows for the selectivity of the MAPK pathways over cellular functions. There are three types of MAPKs known as extracellular signal regulated kinases (ERK), p38 MAPK, and c-Jun-N-terminal kinases (JNK) that regulate apoptosis and proliferation [10]. The latter are activated by stress whereas ERKs are mainly activated by mitogens and growth factor signals. On the contrary, the MAPKs are dephosphorylated on their regulatory threonine and tyrosine residues by MAP kinase phosphatases also known as dual-specificity protein tyrosine phosphatases (DUSPs). The actions of the upstream MAPK activators (MKKs) and downstream MAPK inactivators (MKPs) sets the balance of the cellular outcome of downstream MAPK signaling [14]. Therefore, small changes in MKP regulation will have a significant impact on the outcome of MAPK signaling. Understanding the response of MAPKs to inflammation in the liver is crucial for future treatment plans regarding these diseases. In this review, we examine current evidence supporting the role of MAPK in hepatic inflammation.

## JNK in Hepatic Inflammation

**Figure 1:**
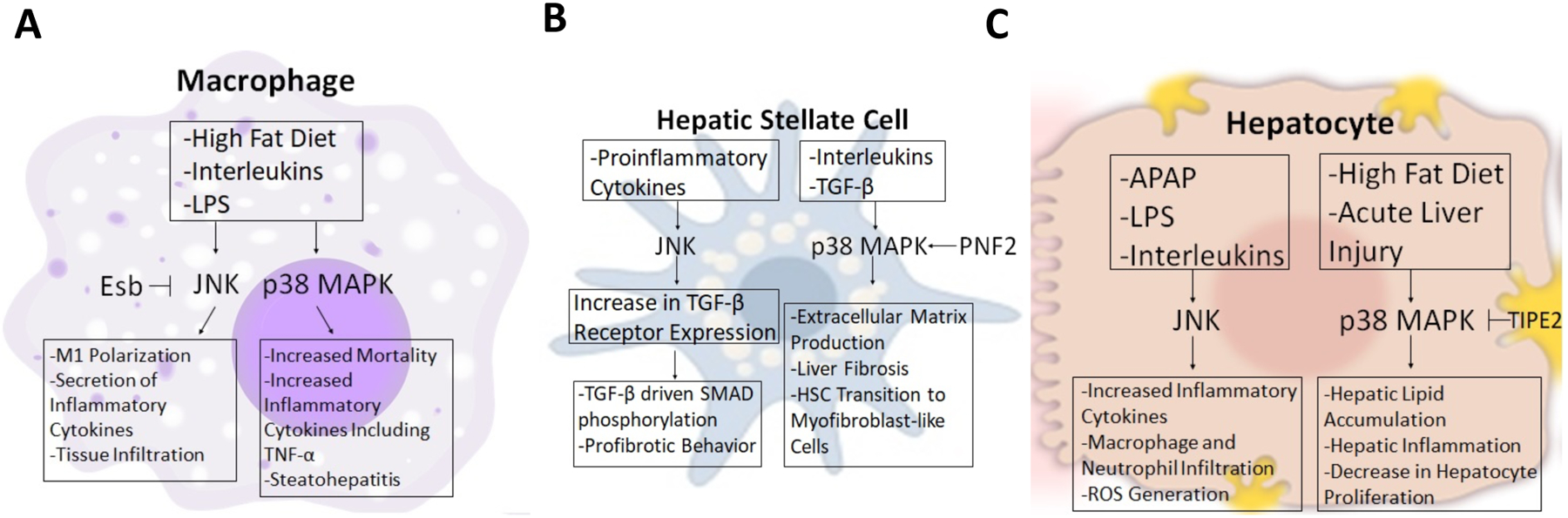
Model for MAPK regulation of inflammation in liver injury. In macrophages **(A)**; HFD activate JNK to promote M1 polarization and secretion of inflammatory cytokines whereas activation of p38 MAPK stimulate secretion of TNFα and steatohepatitis. In hepatic stellate cells (HSC) **(B)**; proinflammatory cytokines activate JNK to stimulate TGF-β expression thereby promoting fibrosis. Interleukins activate p38 MAPK to induce secretion of extracellular matrix and differentiation of HSC to promote hepatic fibrosis. In hepatocytes **(C)**; acetaminophen (APAP) injury activate JNK to stimulate production inflammatory cytokines, macrophage and neutrophil infiltration and formation of reactive oxygen species. HFD and acute liver injury activate p38 MAPK to stimulate hepatic lipid accumulation and inflammation.

The JNK pathway plays a major role in regulating processes including hepatic metabolism and inflammation. There are a total of 13 MAP3Ks that are responsible for activating the JNK pathway, thus allowing it to control a large number of cellular processes [13]. There are three major JNK isoforms including JNK1 (*Mapk8*), JNK2 (*Mapk9*) and JNK3 (*Mapk10*). Since JNK3 is not expressed in the liver, studies of JNKs on hepatic inflammation mainly focused on JNK1 and JNK2. In states of obesity, upregulation of proinflammatory cytokines activates JNK through the action of MKPs that are inactivated as a result of enhanced levels of reactive oxygen species [13,15]. Increased activation of the JNK pathway leads to the expression of inflammatory cytokines and the infiltration of macrophages and neutrophils into the liver generating the inflammatory response (Figure 1A). These effects of JNK are partly mediated by TLR4 and chemokine ligand 16 (CXCL16) as indicated by studies with TLR4 and CXCL16 gene deletion in mice with acetaminophen (APAP) induced liver injury [16]. Another study showed that pharmacological inhibition of JNK (SP600125) protects against acute liver injury (Table 1). The results demonstrated that CXCL16 is a critical regulator of immune response in hepatocytes through the JNK pathways.

In macrophages, downregulation of JNK phosphorylation using natural compounds like Esculentoside B (Esb), inhibited inflammatory response by reducing the secretion of proinflammatory cytokines, iNOS, COX-2, TNF-α, IL-1β, and IL-6 [17]. Another study used chikungunya virus (CHIKV), a mosquito-borne Alphavirus to induce the production of TNF-α in macrophages that were mediated by JNK and p38 MAPK pathways [18]. Others examined fluctuations in glucose concentration on the activation of TLR4-JNK pathway in mediating diabetes-related inflammation in macrophages using THP-1 cells (human monocytes) [19]. The results showed that high glucose concentrations caused activation of the TLR4-JNK pathway leading to inflammation of macrophages on THP-1 cells, suggesting that fluctuations in glucose concentrations mediated by the JNK pathway are detrimental to inflammation of macrophages in diabetes-related vascular diseases.

Alternatively activated M2 macrophages play a critical role in tissue homeostasis. Studies indicate that macrophage scavenger receptor 1 (MSR1) activation drives enhanced JNK signaling, causing a phenotypic switch from anti-inflammatory to pro-inflammatory macrophage behavior [20]. Han et al. used a macrophage-specific JNK1 and JNK2 knockout mouse model to test the role of JNK1 and JNK2 in inflammation and macrophage behavior [21] (Figure 1A). Following high-fat feeding, a macrophage-specific JNK deficient mice displayed lower levels of expression of tissue macrophage marker genes and genes associated with M1 polarization [21]. Genes associated with M2 polarization were higher in JNK macrophage-specific deficient mice. The decrease of M1 tissue macrophages in the liver of high-fat fed knockout mice was associated with a marked reduction in hepatic inflammation compared with the wild type. Macrophage JNK deficiency *in vitro* reduced the chemokine expression of the macrophages under various stimulations [21]. These results suggest that macrophage JNK signaling is necessary for M1 polarization in high-fat fed mice.

There are some studies that begin to unravel the role of JNK in inflammation in human nonalcoholic steatohepatitis (NASH) patients and other liver diseases. Zou et al. found reduced hepatic expression of transcriptional regulator nuclear receptor small heterodimer partner (Nrob2, *SHP*) that was mediated by c-jun-JNK signaling [22]. In macrophages, higher levels of chemokine ligand 2 (CCL2) were observed driven by JNK-dependent suppression of SHP. Fabre et al. used human primary hepatic stellate cells (HSC’s) and the LX2 human HSC cell lines to study fibrosis [23]. When HSCs were co-stimulated with a suboptimal amount TGF-β in conjunction with pro-inflammatory cytokine IL-17A, they found an increase in HSC fibrogenic signaling similar to when optimal concentrations of TGF-β were used [23]. This similarity is due to the increase in TGF-β receptors driven by the IL-17A in a JNK dependent manner. JNK inhibition abrogated the increase in TGF-β receptors driven by IL-17A. This correlated with the loss of SMAD phosphorylation in response to TGF-β signaling, leading to lower expression of pro-fibrotic genes [23] (Figure 1B). These observations demonstrate that inflammation promotes the development of fibrosis.

Collectively, in the liver, phosphorylated JNK was shown to play a pro-inflammatory role in increasing the secretion of inflammatory factors as well as the activation and infiltration of macrophages into the liver tissue. In macrophages, JNK increases the activation of M1 genes and chemokine secretion, suppresses M2 genes, and drives macrophages to infiltrate tissues leading to inflammation. In hepatic stellate cells, JNK was shown to activate fibrogenic genes and stimulate collagen production (Figure 1).

## p38 MAPK in Hepatic Inflammation

p38 MAPKs are proline-directed serine/threonine kinases. p38 MAPKs are encoded in four separate genes in mammalian genomes, including p38α by MAPK14 gene, p38β by MAPK11, p38γ by MAPK12, and p38δ by MAPK13 [24]. p38 MAPKs have been shown to be responsive to various stress stimuli from environmental and intracellular stresses [13,24–26]. Several studies have demonstrated the role of hepatic p38 MAPK in the control of hepatic inflammation.

Recently, one study investigated the role of p38α in a high-fat diet (HFD)-induced fatty liver. In this study, hepatocyte-specific deletion of p38α exhibited greater steatosis and higher levels of hepatic triglyceride after 3 months of HFD feeding by reducing expression of genes involved in fatty acid oxidation, including fibroblast growth factor 21, carnitine palmitoyltransferase I, acyl-CoA oxidase1, and peroxisome proliferator-activated receptor alpha [27]. Further, oxidative stress and inflammation increased in mice with hepatocyte-specific deletion of p38α [27]. In contrast, another study demonstrated that a tumor necrosis factor ameliorates lipid accumulation and inflammation in hepatocytes with HFD-induced hepatic steatosis by inhibiting p38 MAPK activity [28]. This study evaluated the role of tumor necrosis factor-α-induced protein 8-like 2 (TIPE2), which is expressed in immune cells and negatively regulates inflammatory diseases. They found that p38 MAPK activity was significantly attenuated in hepatocytes with TIPE2-overexpression, leading to protective effects against non-alcoholic fatty liver disease (NAFLD) [28]. Protection of hepatocytes following acute liver injuries plays an important role in preventing chronic liver injury and hepatic fibrosis [29,30] (Figure 1C). p38α has been shown to act as a negative regulator of hepatocyte proliferation in response to acute liver injuries [31–33]. Phosphorylation of p38 MAPK is significantly increased in the liver following hepatotoxin-induced acute liver injury [34] and in a model of dengue virus-induced liver injury inhibition of p38 MAPK (SB203580) improved hematological indices, histopathology and cell death (Table 1).

Growing evidence suggests the important role of p38 MAPK in hepatic macrophages, which play an important role in the hepatic repair after liver injury. Macrophage p38α deficient mice display decreased mortality and relieved drug-induced hepatotoxicity with resistance to apoptosis, accelerated regeneration, and decreased cytokine production [35]. Macrophage lacking p38α is also resistant to the development of steatohepatitis in response to high-fat/high-cholesterol diet (HFHC) [36]. The primary hepatocytes derived from macrophage p38α-deficient mice showed decreased steatosis and inflammatory damage through decreased secretion of pro-inflammatory cytokines, including TNF-α, CXCL10, and IL-6, which regulate M1 macrophage polarization [36] (Figure 1A).

During liver damage, hepatic stellate cells (HSCs) are highly responsive to proinflammatory cytokines [37]. p38 MAPK has been involved in the activation of HSCs, which results in the production of extracellular matrix and liver fibrosis [38]. One study demonstrated that the RING finger protein (PNF2), which is abnormally expressed in hepatocellular carcinoma, is highly upregulated in fibrotic liver tissue and knockdown of PNF2 inhibits phosphorylation of p38 MAPK in HSCs in response to transforming growth factor-β1 (TGF-β1), leading to a reduction of liver fibrogenesis [38] (Figure 1B). The activity of p38 MAPK is also involved in the transition of HSCs to myofibroblast-like cells following liver injury. It has been demonstrated that phosphorylation levels of p38 MAPK significantly increased in HSCs treated with IL-6, which is known as an inducer of HSC activation in response to hepatic inflammation [39].

Together, p38 MPAK appears to play an important role in contributing to the progression of hepatic inflammation in response to various hepatic cellular stresses. Future studies will require to identifying p38 MAPK-mediated substrates in hepatocytes and hepatic stellate cells using specific deletion of p38 MAPK (Figure 1).

## ERK in Hepatic Inflammation

The extracellular-regulated kinase (ERK) pathway is the most widely characterized among members of MAPK family. The ERK family consists of ERK1–8. Among them ERK1 and ERK2 are best the characterized isoforms [10,11]. They participate in the Ras-Raf-MEK-ERK signal transduction cascade, which is important in regulating functions such as cell growth, cell adhesion, cell cycle progression, cell migration, cell proliferation, and cell survival [11,40]. When the ERK1 and ERK2 isoforms are activated, they can translocate to the nucleus and activate several transcription factors such as, c-Fos, ATF-2, ELK-1, c-Jun, c-Myc, and Ets-1 [41,42]. ERK pathway has been shown to play an important role in mediating hepatic inflammation.

Recently, ERK pathway has been associated with liver injury due to the overuse of acetaminophen. Typically, acetaminophen can be metabolized by glucuronidation and sulfuration in hepatocytes and only a small amount of toxic N-acetyl-p-benzoquinone imine (NAPQI) is activated by cytochrome P450 [5]. This toxic NAPQI byproduct conjugates with glutathione (GSH). In the cases of acetaminophen overdose, GSH cannot handle the amount of toxic NAPQI resulting in mitochondrial dysfunction and oxidative stress which ultimately leads to hepatocyte necrosis through the ERK pathway [5]. The authors found that escin can induce protective effects against acetaminophen related liver injury as a consequence of anti-inflammatory mechanism and ERK signaling pathway inhibition. This study supports the notion that acetaminophen overdose results in an innate immune response that attributes to hepatocyte death. DAMPs are released during hepatocyte necrosis and recognized by Kupffer cells and macrophages through toll-like receptors. The macrophages then release CXC chemokines that aid in recruitment of neutrophils and monocytes [5]. The myeloid differentiation primary response gene 88 (MyD88) plays an important role in inflammation. Mice lacking MyD88, specifically in hepatocytes exhibit fatty liver and inflammation [43]. To assess inflammation sensitivity, mice lacking MyD88 were given acute LPS injection resulting in increased inflammation in MyD88 knockout mice compared to wildtype [43]. Consistent with this, the hepatic mRNA levels of TNF-α, IL-6 and IL-1β and 25-hydroxycholesterol levels were increased in MyD88 knockout mice. Also, in the absence of MyD88, the mice showed impaired bile acid and oxysterol metabolism. ERK activity was reduced in MyD88 knockout mice suggesting that these observations could be mediated by the ERK pathway [43].

The role of hepatic stellate cells was examined using mice with disrupted interleukin 11 receptor subunit alpha 1 gene fed a high-fat methionine and choline-deficient or western diet with liquid fructose to induce steatohepatitis [44]. Stimulation of hepatic stellate cells with cytokines caused these cells to produced IL-11 leading to the activation of ERK phosphorylation increased markers for hepatic fibrosis and inflammation [44]. Many studies have demonstrated that hepatic myofibroblasts (MFs) play a role in developing liver fibrogenesis and are modulated by ERK signaling pathway. These hepatic myofibroblasts are known to be derived from hepatic stellate cells (HSC), portal fibroblasts, bone-marrow derived cells like mesenchymal stem cells (MSCs) and fibrocytes [45]. It is known that once activated, hepatic MFs are able to synthesize and release pro-inflammatory mediators, such as chemokines CCL12 and CCL21. Also, they are able to activate IL-1b by activating inflammasome NLRP3 [46]. Foglia et al. showed that pentoxifylline resulted in ERK inhibition, which indicated positive anti-fibrogenic activity, use of N-acetyl cysteine and curcumin resulted in cell cycle arrest at the G1 phase by regulating ERK [47]. Also, hepatic myofibroblasts when exposed to LPS, Angiotensin II, TNF, and interferon-gamma, expressed reactive oxygen species such as NOX2, NOX1, and NOX4 which affects ERK pathway sustaining pro-inflammatory responses as well as proliferation, ECM synthesis, and migration [47]. Other studies inhibited ERK (U0126) pathway in a model of LDLR knockout and observed reduced triglyceride production and decreased atherosclerosis (Table 1).

Together, these observations suggest that LPS and overnutrition activates the ERK pathway thereby promoting the development of inflammation and fibrosis. However, more studies are required to define the ERK substrates mediating these effects.

## Role of MAPK in Liver Regeneration and Hepatitis Viruses

Activation of the MAPK pathway represents an important and fundamental mechanism through which hepatocyte function, hepatocyte life and death is regulated. One of the most studied models of cell, organ and tissue regeneration is liver regeneration following partial hepatectomy (PH). Two different mice models of hepatic steatosis demonstrated obstructed hepatocyte proliferation following PH that was associated with reduced liver to body weight ratio and cyclin A expression [54]. Administration of growth hormone activated hepatic ERK signaling leading to upregulation of Myc, FOS and JUN and improved hepatocyte proliferation compared with control mice [54]. Backes et al. showed that stenosis-induced reduced portal vein blood flow negatively affected liver regeneration [55]. Rats administered either insulin or tacrolimus exhibited enhanced liver mass and markers of proliferation – IL-6 and Ki67 compared with the group that had simple portal vein stenosis [55]. These studies demonstrate that MAPK pathway is necessary for the mitogenic actions of growth hormone and insulin during liver regeneration. Hepatitis B virus (HBV) is a major cause of many chronic liver diseases including liver cirrhosis and the hepatocellular carcinoma [56]. Yang et al., demonstrated that during early infection phase HBV activates the phosphorylation of p38 MAPK that promoted HBV replication [57], and induction of HoxA10 during late HBV replication reduce activation of p38 MAPK thereby attenuating HBV replication [57]. Takaki et al., examined the mechanism of apoptosis in hepatitis C virus (HCV)-infected hepatocytes [58]. They found increased apoptosis in HCV infected hepatocytes that was partly mediated by enhanced phosphorylation of JNK [58]. These findings demonstrate that p38 MAPK and JNK play an important role in the progression of hepatitis viral infections and other metabolic diseases associated with chronic hepatitis.

## Conclusion

Multiple studies provide evidence that in the liver, JNK phosphorylation plays a proinflammatory role in increasing the secretion of inflammatory factors as well as the activation and infiltration of macrophages into the liver tissue. In macrophages, JNK increases the activation of M1 genes and chemokine secretion, suppresses M2 genes, and drives macrophages to infiltrate tissues leading to inflammation. In hepatic stellate cells, JNK activates fibrogenic genes and stimulates collagen production. p38 MPAK plays an important role in contributing to the progression of hepatic inflammation in response to various hepatic cellular stresses. Future studies will require to identify p38 MAPK-mediated substrates in hepatocytes and hepatic stellate cells using specific deletion of p38 MAPK. Evidence for the role of the ERK pathway in hepatic inflammation and fibrogenesis and more studies are required to define the ERK substrates mediating these effects.

## Figures and Tables

**Table 1:** Recent Studies Using Pharmacological Inhibitors/Mediators to Investigate the Role of MAPKs in Hepatic injury/Disease.

Pharmacological Inhibitors/mediators of MAPK	Hepatic Injury/Disease model	Phenotype	MAPK Expression	References
SB203580 (p38 MAPK)	Mouse model of dengue virus-induced liver injury	Improved hematological parameters, histopathology and apoptosis. Reduced expression of TNFα, IL-6 and IL-10	No change in P38 MAPK phosphorylation but reduced phosphorylation of both MAPKAPK2 and ATF-2	[48]
U0126 (ERK MAPK)	Spred-2 knockout mice fed high fat diet	Decreased inflammatory cytokine response and adipose inflammation	Inhibition of the MEK/ERK pathway	[49]
U0126 (ERK MAPK)	LDLR knockout mice fed high fat diet	Reduced atherosclerosis, triglyceride overproduction, de novo lipogenesis	Inhibition of MEK/ERK pathway	[50]
SP600125 (JNK MAPK)	Acetaminophen-induced liver injury in Nrf2 knockout mice	Upregulation of antioxidant response element genes and inhibited Nrf2 phosphorylation	Inhibition of JNK phosphorylation	[51]
SP600125 (JNK MAPK)	CCl4 or acetaminophen-induced liver injury in JNK1 and JNK1/2 hepatocyte specific knockout mice	Protection from acute liver injury	Inhibition of JNK phosphorylation	[52]
SP600125 (JNK MAPK)	Mice model of portal vein ligation for staged hepatectomy	Downregulation of hepatocellular regeneration and Indian Hedgehog signaling	Inhibition of JNK1 phosphorylation	[53]
SB203580 (p38 MAPK), SP600125 (JNK MAPK), PD98059(ERK MAPK)	Diethylnitrosamine (to induce fibrosis) and salvianolic acid B (to alleviate fibrosis) were injected into mice intraperitoneally	Alleviated liver fibrosis, decreased fibrotic marker protein expression Collagen 1, TGFβ1, a-SMA) and improved histopathological features	Decreased phosphorylation of ERK1/2, JNK1/2, and P38 MAPK	[54]
